# Health Coaching Reduces HbA1c in Type 2 Diabetic Patients From a Lower-Socioeconomic Status Community: A Randomized Controlled Trial

**DOI:** 10.2196/jmir.4871

**Published:** 2015-10-05

**Authors:** Noah Wayne, Daniel F Perez, David M Kaplan, Paul Ritvo

**Affiliations:** ^1^ School of Kinesiology & Health Science Faculty of Health York University Toronto, ON Canada; ^2^ North York General Hospital Department of Family & Community Medicine University of Toronto Toronto, ON Canada

**Keywords:** diabetes mellitus, type 2, health coaching, mHealth, telehealth, randomized controlled trial, RCT

## Abstract

**Background:**

Adoptions of health behaviors are crucial for maintaining good health after type 2 diabetes mellitus (T2DM) diagnoses. However, adherence to glucoregulating behaviors like regular exercise and balanced diet can be challenging, especially for people living in lower-socioeconomic status (SES) communities. Providing cost-effective interventions that improve self-management is important for improving quality of life and the sustainability of health care systems.

**Objective:**

To evaluate a health coach intervention with and without the use of mobile phones to support health behavior change in patients with type 2 diabetes.

**Methods:**

In this noninferiority, pragmatic randomized controlled trial (RCT), patients from two primary care health centers in Toronto, Canada, with type 2 diabetes and a glycated hemoglobin/hemoglobin A1c (HbA1c) level of ≥7.3% (56.3 mmol/mol) were randomized to receive 6 months of health coaching with or without mobile phone monitoring support. We hypothesized that both approaches would result in significant HbA1c reductions, although health coaching with mobile phone monitoring would result in significantly larger effects. Participants were evaluated at baseline, 3 months, and 6 months. The primary outcome was the change in HbA1c from baseline to 6 months (difference between and within groups). Other outcomes included weight, waist circumference, body mass index (BMI), satisfaction with life, depression and anxiety (Hospital Anxiety and Depression Scale [HADS]), positive and negative affect (Positive and Negative Affect Schedule [PANAS]), and quality of life (Short Form Health Survey-12 [SF-12]).

**Results:**

A total of 138 patients were randomized and 7 were excluded for a substudy; of the remaining 131, 67 were allocated to the intervention group and 64 to the control group. Primary outcome data were available for 97 participants (74.0%). While both groups reduced their HbA1c levels, there were no significant between-group differences in change of HbA1c at 6 months using intention-to-treat (last observation carried forward [LOCF]) (*P*=.48) or per-protocol (*P*=.83) principles. However, the intervention group did achieve an accelerated HbA1c reduction, leading to a significant between-group difference at 3 months (*P*=.03). This difference was reduced at the 6-month follow-up as the control group continued to improve, achieving a reduction of 0.81% (8.9 mmol/mol) (*P*=.001) compared with a reduction of 0.84% (9.2 mmol/mol)(*P*=.001) in the intervention group. Intervention group participants also had significant decreases in weight (*P*=.006) and waist circumference (*P*=.01) while controls did not. Both groups reported improvements in mood, satisfaction with life, and quality of life.

**Conclusions:**

Health coaching with and without access to mobile technology appeared to improve glucoregulation and mental health in a lower-SES, T2DM population. The accelerated improvement in the mobile phone group suggests the connectivity provided may more quickly improve adoption and adherence to health behaviors within a clinical diabetes management program. Overall, health coaching in primary care appears to lead to significant benefits for patients from lower-SES communities with poorly controlled type 2 diabetes.

**Trial Registration:**

ClinicalTrials.gov NCT02036892; http://clinicaltrials.gov/ct2/show/NCT02036892 (Archived by WebCite at http://www.webcitation.org/6b3cJYJOD)

## Introduction

### Overview

The type 2 diabetes mellitus (T2DM) epidemic is an increasing economic and personal health burden that could be cost-effectively addressed with health coach (HC) interventions, assisted by mobile phone technologies [1]. HC interventions target health behavior changes aligned with self-determined goals leading to improved physical and mental health outcomes [2]. Chronic medical conditions are targeted when health behaviors adopted by patients can significantly reduce risks of worsened disease and disease complications [3].

Amid promising reports of computer and mobile phone-assisted health interventions [4], a dearth of studies focus on which types of personal interactions combine most effectively with current technologies. Prior to this randomized controlled trial (RCT), we codeveloped, with NexJ Systems Inc, mobile phone software for logging health data (eg, blood glucose, blood pressure, mood, energy, and pain) and related activities (eg, exercise, diet, and stress) using secure, cloud-based storage. The software permits innovative comonitoring of client behaviors (eg, photographing meals) and transmission of reminder messages encouraging activation and adherence. As the HC reviews participant activities in real-time experience, these immediately responsive communications can prevent relapse and/or assist relapse recovery, as demonstrated in a pilot study [3].

Internet-based interventions have demonstrated significant improvements in glucoregulation in T2DM patients, as exemplified in a cluster RCT undertaken by Quinn et al (2011) where 4 different intensity levels of Internet-based support were compared; significant between-group differences in reduced glycated hemoglobin/hemoglobin A1c (HbA1c) were found when the most intense intervention (*P*<.001) was compared to usual care. This intervention consisted mainly of automated messages prompted by patient entries (eg, self-assessed blood glucose) and the patients studied were all health insured, after exclusion of the noninsured population that is often associated with lower socioeconomic status (SES), higher T2DM prevalence, and poorer glucose control [5,6]. In contrast, our intervention included a high proportion of lower-SES patients as all Ontario residents are able to access essential health services free of charge via the Ontario Health Insurance Program (OHIP). Our trial focused on supporting participants in surmounting the additional challenges confronted by lower-SES community residents, such as poor neighborhood walkability [7] and elevated consumption of energy-dense/nutrient-poor foods [8]. Failure to surmount these challenges often leads to an increased longitudinal use of health care resources due to more reactive use combined with poorer health status [9]. A further contrast was that our study was based on assessing HC interactions, with and without mobile phone-based support.

Another more recent trial compared a mobile phone-based, self-management system with and without telephone-based health coaching in improving HbA1c levels, with a usual care control group. Both intervention groups accessed a mobile phone-based self-management system that enabled users to track blood glucose, diet, physical activity, and personal goals. The most intensive intervention group received health coaching delivered by a diabetes specialist nurse for the first 4 months of the 12-month trial, with a total of five 20-minute phone contacts. Results indicated no significant between-group or within-group HbA1c differences [10]. The intensity of this HC intervention—five 20-minute phone contacts—was considerably lower than the levels applied in this study.

The importance of lowering HbA1c and improving glucoregulation in T2DM patients cannot be overemphasized as HbA1c is a robust indicator of complication risks and a widely accepted tool for T2DM diagnosis [11]. Without proper management, patients with T2DM are at increased risk for debilitating complications, particularly stroke [12], neuropathy leading to amputation and blindness [13], and death [14]. HbA1c reductions have been associated with carbohydrate control [15], vigorous exercise [16], and medication adherence [17].

While the economic pressures of funding interventions motivate technological developments that can, in part or whole, replace personal counseling interventions, studies that compare different HC intensities combined with different technologies are necessary to determine optimal proportions. The usefulness of such studies is exemplified by Nundy et al (2013) who assessed a mobile phone-based, automated text messaging and counselling intervention with type 2 diabetes patients. In a quasi-experimental, two-group, pre-/post-design, intervention participants appeared to be 8.8% less costly *during* the 6-month intervention, than during the 6 months preceding intervention engagement. These participants also reduced their HbA1c by 0.7% leading to other potential longitudinal cost savings not yet evaluated [18]. Because all were participants in the University of Chicago employee health plan, relevant health care costs were accessed and compared. Once again, our study differs in that our sample included unemployed individuals who would have been ineligible for the health plan which this previous study relied on.

The purpose of increasing the frequency and intensity of any health behavior in T2DM patients is improved glucoregulation, which directly and/or indirectly influences health-related quality of life (HRQOL). It is important to assess HRQOL outcomes independently through secondary RCT analyses, as improvements in physical health that are not associated with positive changes in quality of life are not likely to be sustainable. We used the comparison analyses of this study and, additionally, the qualitative analyses of semistructured interviews found in a companion study [19].

### Objective

Based on data from a previous pilot trial, this noninferiority pragmatic RCT tested the effectiveness of a mobile phone-based health coaching protocol, versus one without mobile phone support, in reducing the HbA1c of patients with T2DM from a lower-SES community.

## Methods

### Overview

This pragmatic RCT proceeded with a 1:1 allocation ratio. Participants were recruited from 2 primary health clinics in Toronto, Canada, between March 2012 and October 2013. The populations served were from a lower-income neighborhood (90% of participants) and a midlevel-SES community (10% of participants). Patients were eligible for participation if diagnosed with T2DM, if they had an HbA1c ≥ 7.3% (56.3 mmol/mol) measured within 1 month of consent, and if they were under 70 years of age. Following pragmatic trial guidelines, there were no additional exclusion criteria (eg, no exclusion of individuals with psychiatric diagnoses). All study protocols were approved by the Research Ethics Boards at York University, North York Family Health Team, and North York General Hospital. This RCT was registered with ClinicalTrials.gov (NCT02036892) and reported following CONSORT-EHEALTH statement guidelines [20].

Recruitment was undertaken through phone contacts with eligible individuals identified via clinic electronic medical records. Additional recruitment assistance was obtained from associated diabetes education programs, primary care physicians, and locally practicing endocrinologists.

When participants agreed to an initial meeting to discuss the study, their HbA1c findings were verified, the study protocol was explained, and informed consent was obtained. Eligible patients then completed demographic and psychometric questionnaires and were randomized. Table 1 shows the baseline characteristics of the participants.

**Table 1:** Baseline characteristics (as per study protocol).

Baseline characteristics		Whole sample (n=97), mean (SD) or n (%)^a^	Intervention group (n=48), mean (SD) or n (%)^a^	Control croup (n=49), mean (SD) or n (%)^a^
Age in years, mean (SD)		53.2 (11.3)	53.1 (10.9)	53.3 (11.9)
**Location, n (%)**				
	Site #1: BCCHC^b^	90 (93)	46 (96)	44 (90)
	Site #2: NYFHT^c^	7 (7)	2 (4)	5 (10)
**Gender, n (%)**				
	Male	27 (28)	17 (35)	10 (20)
	Female	70 (72)	31 (65)	39 (80)
**Ethnicity, n (%)**				
	First Nations	1 (1)	0 (0)	1 (2)
	Black: African	5 (5)	3 (6)	2 (4)
	Black: Caribbean	39 (40)	19 (40)	20 (41)
	Caucasian	26 (27)	12 (25)	14 (29)
	Hispanic	9 (9)	4 (8)	5 (10)
	South Asian	4 (4)	3 (6)	1 (2)
	South East Asian	4 (4)	2 (4)	2 (4)
	West Indian	6 (6)	3 (6)	3 (6)
	Other	3 (3)	2 (4)	1 (2)
**Highest education level achieved, n (%)**				
	Less than high school	22 (23)	10 (21)	12 (24)
	High school diploma	35 (36)	17 (35)	18 (37)
	College or vocational training	25 (26)	11 (23)	14 (29)
	University degree	12 (12)	8 (17)	4 (8)
	Not disclosed	3 (3)	2 (4)	1 (2)
**Employment, n (%)**				
	Unemployed	35 (36)	16 (33)	19 (39)
	Student	4 (4)	3 (6)	1 (2)
	Part time	6 (6)	1 (2)	5 (10)
	Full time	25 (26)	13 (27)	12 (25)
	Retired	11 (11)	6 (13)	5 (10)
	Self-employed	9 (9)	6 (13)	3 (6)
	Work in home (eg, take care of children)	4 (4)	2 (4)	2 (4)
	Not disclosed	3 (3)	1 (2)	2 (4)
**Income in Can $, n (%)**				
	$0-$9999	21 (22)	9 (19)	12 (25)
	$10,000-$25,000	23 (24)	10 (21)	13 (27)
	$25,000-$50,000	20 (21)	12 (25)	8 (16)
	$50,000-$75,000	9 (9)	3 (6)	6 (12)
	$75,000 and higher	5 (5)	4 (8)	1 (2)
	Not disclosed	19 (20)	10 (21)	2 (4)
**Car access, n (%)**				
	Owns a car	35 (36)	19 (40)	16 (33)
	Has access to car	12 (12)	9 (19)	3 (6)
	No access to car	48 (50)	19 (40)	29 (59)
	Not disclosed	2 (2)	1 (2)	1 (2)

^a^Percentages may not add up to 100% due to rounding.

^b^Black Creek Community Health Centre (BCCHC).

^c^North York Family Health Team (NYFHT).

### Intervention

**Figure 1:**
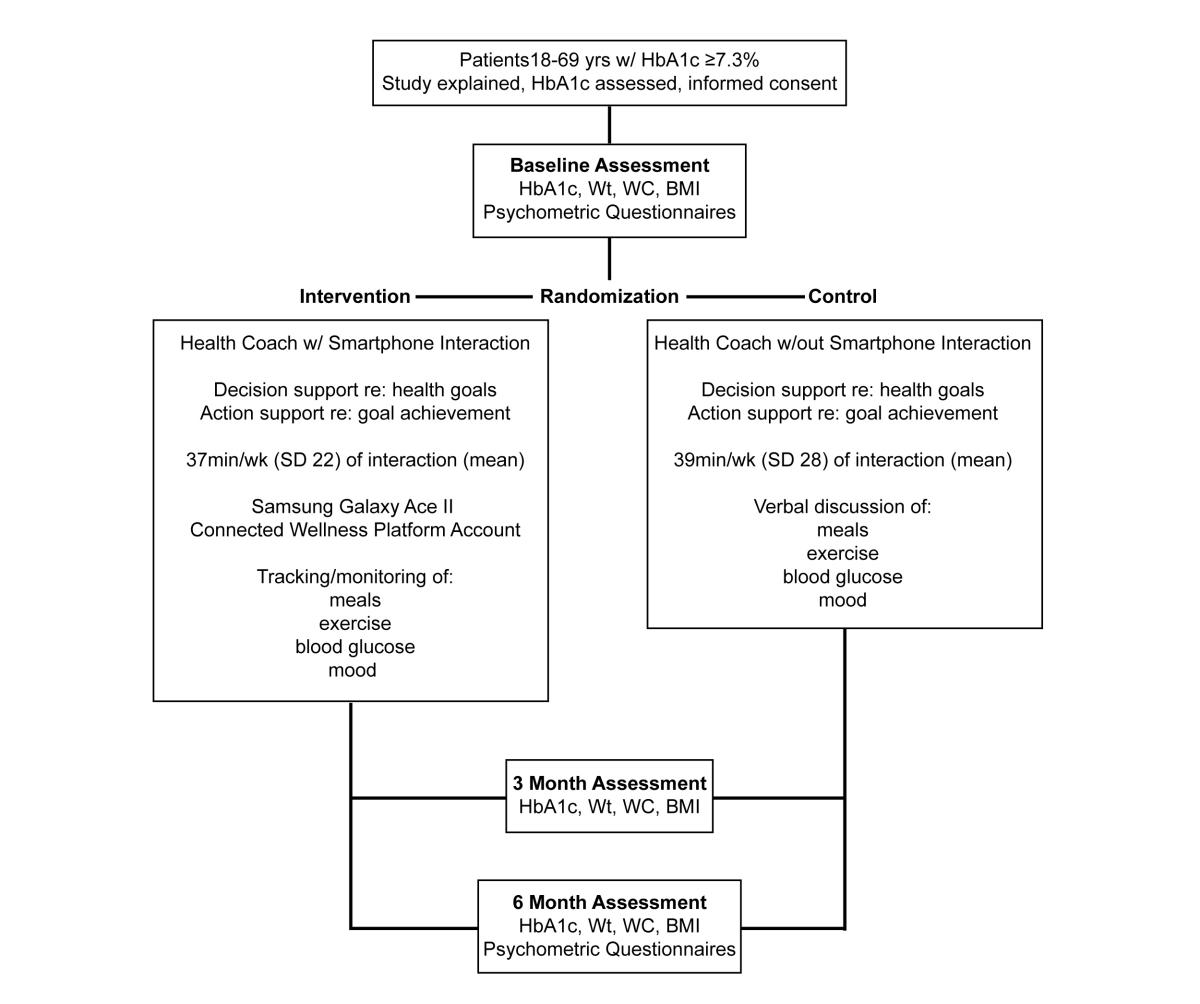
Experimental design and timing of data collection.

The HC intervention extended for 6 months from the date of consent (Figure 1) following a behavior-change curriculum designed by 2 study authors (PR and NW) at York University that incorporated feedback from the prior pilot study [3]. In the intervention, a health coach was defined as a behavior-change counselling specialist with expertise in chronic disease management and evidence-based theory adapted for disease state, SES, and ethnocultural backgrounds. With HC assistance, clients determined health-related goals and monitored daily progress. The HC comonitored the client’s mobile phone input and directed immediate attention (on a 24-hour/day and 7-day/week basis) to episodes of desirable progress, relapse, and resistance. The HC protocol has been manualized, emphasizing those situations observed to frequently arise when behavior change is addressed in T2DM-affected individuals.

Eligible participants were randomized to the respective study groups (with and without mobile phone support), with HCs in both groups guiding participants in planning and reaching health targets aimed at reducing HbA1c. Efforts focused primarily on increasing exercise (frequency, duration, intensity) and modifying diet to reduce carbohydrate intake. Additional goals emphasized stress management, medication adherence, and effective communication with primary care physicians and, generally, within the health system.

Six HCs intervened with experimental and control group participants. These individuals held bachelor’s degrees in kinesiology and health science and/or were graduate students in the School of Kinesiology and Health Science at York University. Five HCs were certified exercise physiologists and one was a certified personal trainer, all certified by the Canadian Society for Exercise Physiology (CSEP). All attended weekly seminars prior to and throughout the trial where they received training in the HC curriculum by the lead investigator (PR). HCs also participated in weekly team meetings led by the study coordinator (NW) where they discussed applications of behavior theory in specific strategies for each participant.

The Black Creek Community Health Centre (BCCHC) concurrently provided the Exercise Education Program (EEP) to all community members (free of charge) that featured exercise prescription, monitoring, and adherence support. Participants were monitored on both an individual and group basis by trainers during exercise sessions and patients with T2DM were provided with special blood glucose testing before and after each exercise session. The program included group exercise classes, resistance training with weights and bands, and cardiovascular exercise using a treadmill and stationary bicycles. Both intervention and control group participants had EEP access for the trial duration.

### Intervention Group

**Figure 2:**
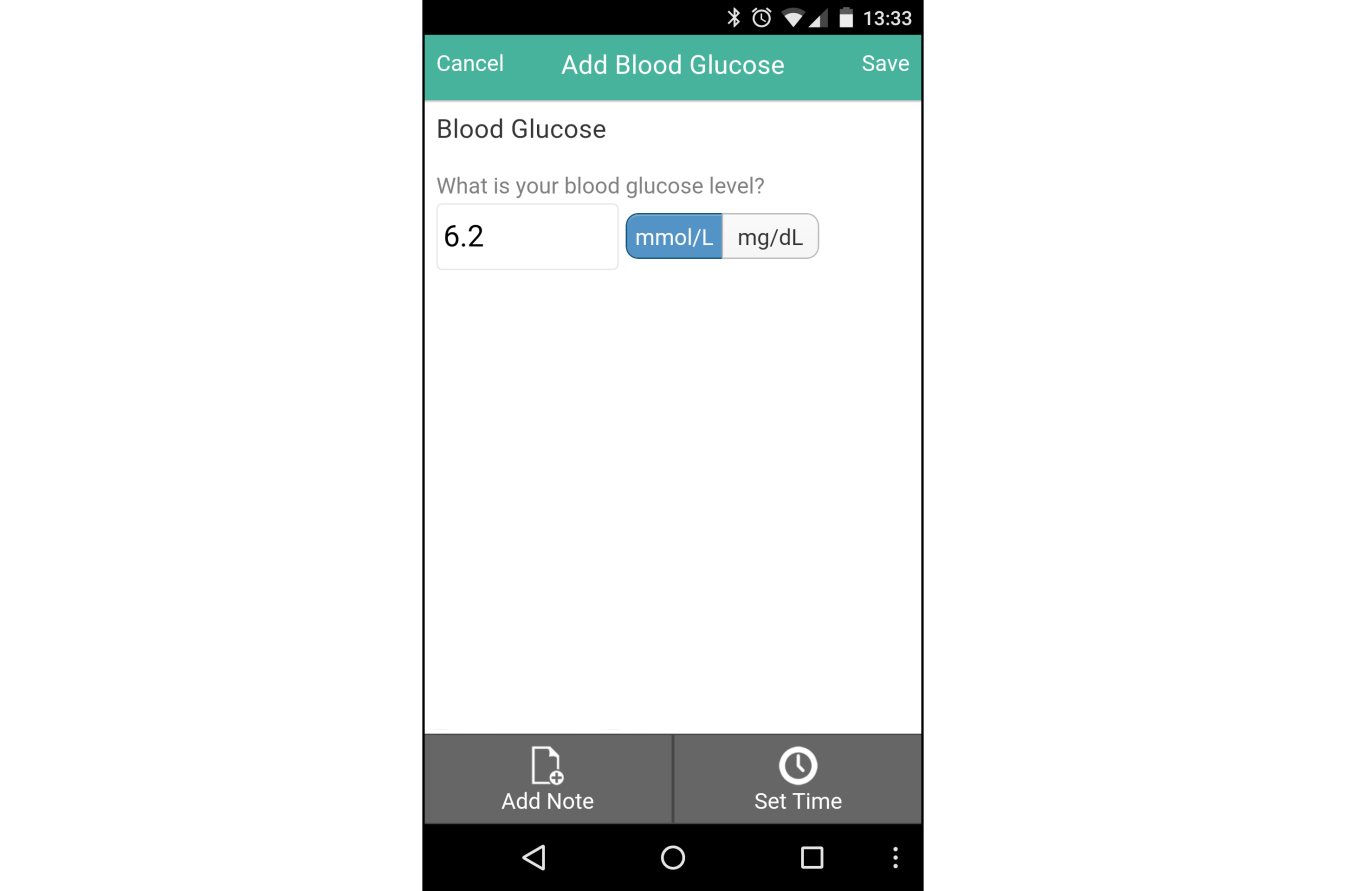
Screenshot of blood glucose tracker on the Connected Wellness Platform from NexJ Systems, Inc.

**Figure 3:**
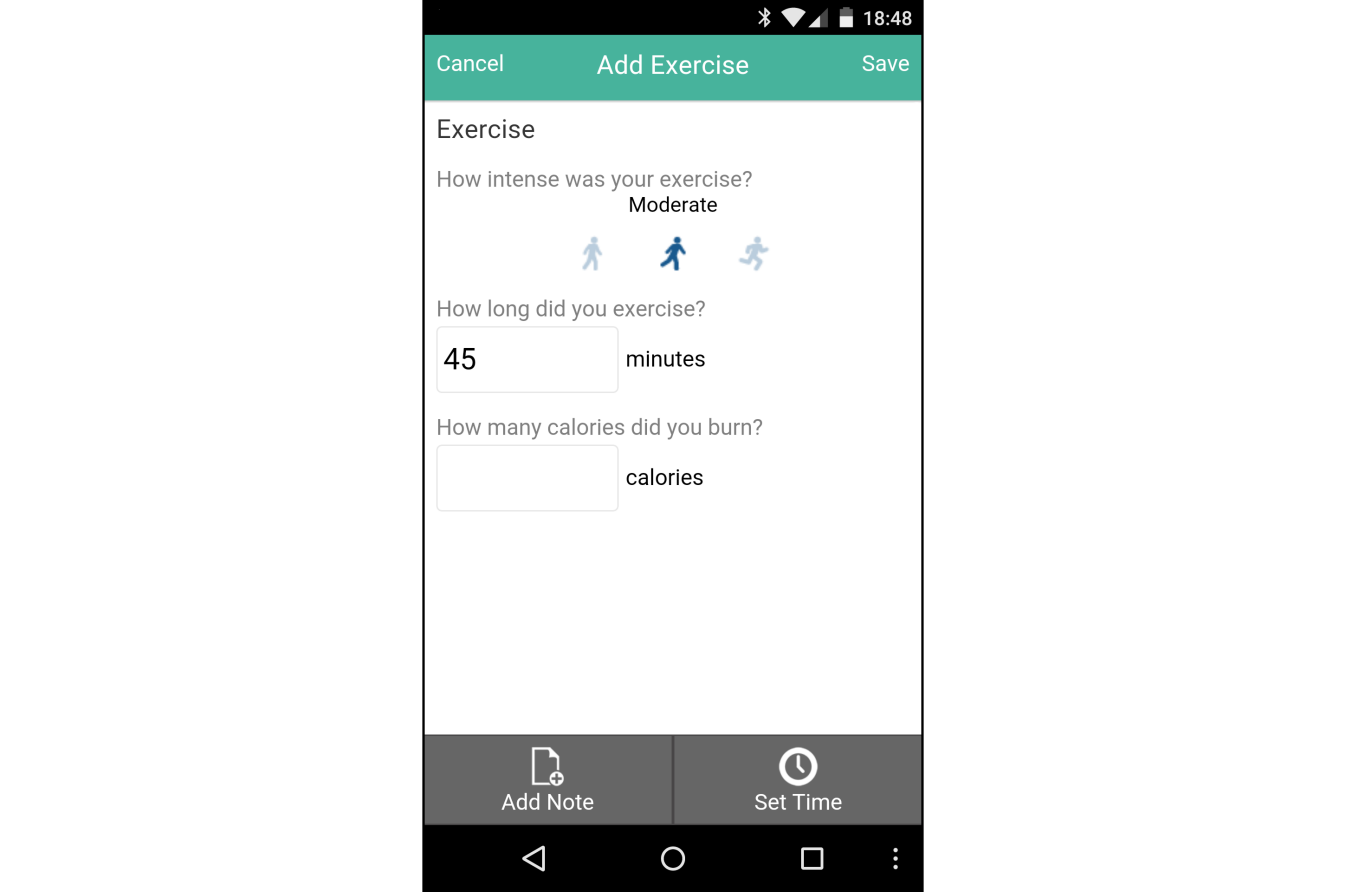
Screenshot of exercise tracker on the Connected Wellness Platform from NexJ Systems, Inc.

**Figure 4:**
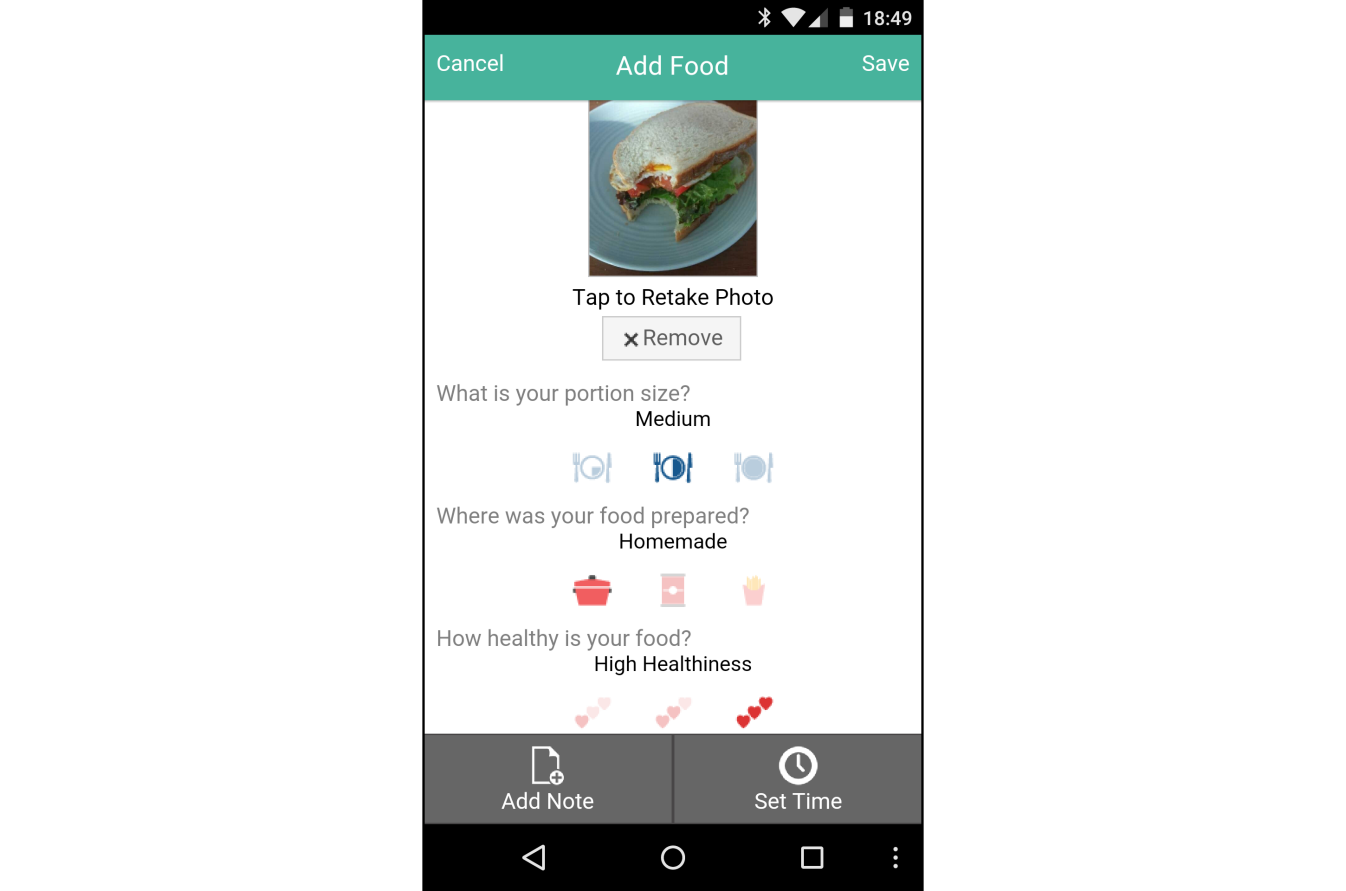
Screenshot of food tracker on the Connected Wellness Platform from NexJ Systems, Inc.

**Figure 5:**
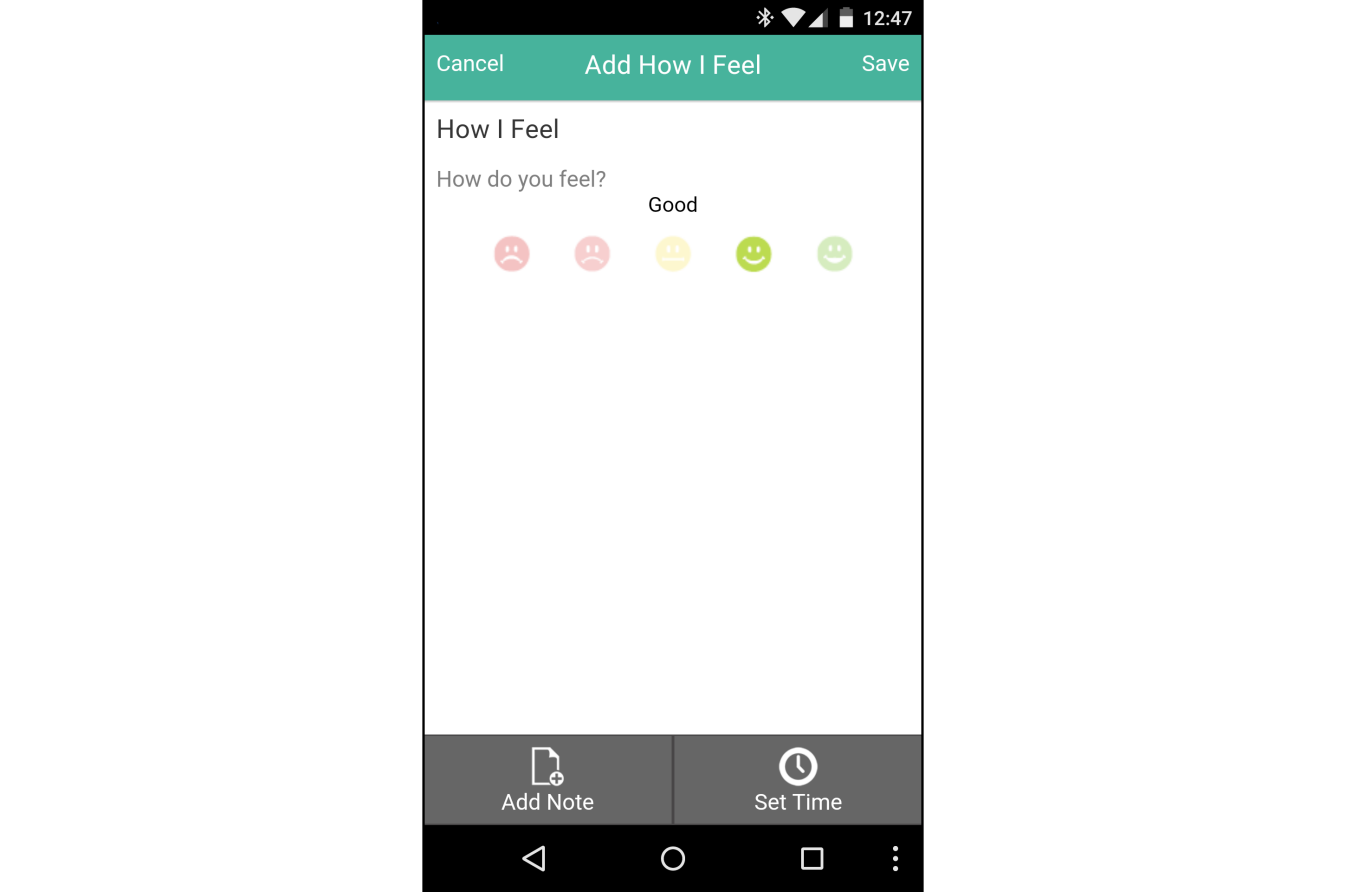
Screenshot of mood tracker on the Connected Wellness Platform from NexJ Systems, Inc.

The intervention group was provided with a Samsung Galaxy Ace II mobile phone running Google Android Ice Cream Sandwich (Android 4.0.2) for the study intervention period, with a data-only carrier plan. They were also provided a user account with the Connected Wellness Platform (CWP) provided by NexJ Systems, Inc [21], which supported participants in health-related goal setting and progress monitoring. Participants could track key metrics, notably blood glucose levels (Figure 2), exercise frequency/duration/intensity (Figure 3), food intake (via photo journaling) (Figure 4), and mood (Figure 5). They could communicate with their health coach at any time in the 24-hour cycle via secure messaging, scheduled phone contact, and/or during in-person meetings. The mean total contact (for all these activities) was 38 minutes/week (SD 25). All health data entered by participants into the CWP were immediately visible to health coaches through a secure, Web-accessible portal. Although participants were encouraged to use the system daily, individual usage patterns varied. Participant data and software-enabled communication required two-way, certificate-based authentication and passwords that were stored in encrypted columns. The CWP exceeds Canadian privacy standards for software carrying health information. Based on patient goals, HCs used the 24-hour/day logging function to guide healthy lifestyle choices, while providing support when clients diverged from intended health goals and routines.

### Control Group

Control group participants received HC support in selecting and progressing toward goals without access to a (study-provided) mobile phone or the CWP software. Control group participants accessed the EEP, as did the intervention group participants for the study duration, in addition to in-person meetings and health coach phone contacts.

### Primary Outcome

The primary outcome was the difference between intervention and control group means of HbA1c levels from baseline to 6 months. Intention-to-treat and per-protocol analyses were both undertaken and are presented below. HbA1c levels were assessed by physician requisition or, when unobtainable, by a point-of-care HbA1c analyzer (Siemens DCA Vantage 3000) which has met performance criteria in efficacy trials [22] and has been employed in comparable research trials [10,23]. To ensure consistency, the type of HbA1c collection at baseline was the same at follow-up sessions. While the 3-month assessment allowed an evaluation of trends, the 6-month assessment was used as the primary outcome. Measures of blood work were accepted within 4 weeks of the 3- and 6-month measurement intervals providing flexibility for participant schedules and physician requisitions.

### Secondary Outcomes

Differences between HbA1c mean levels within groups were also analyzed. Additional outcomes included anthropometric measurements for weight (kg), body mass index (BMI) (kg/m^2^), and waist circumference (cm) collected at baseline and 6-month time points. Changes in psychometric assessments at baseline and 6 months were analyzed using the Satisfaction with Life Scale [24], the Hospital Anxiety and Depression Scale [25], the Positive and Negative Affect Schedule [26], and the Short Form Health Survey-12 (SF-12) [27]. All measures were obtained on-site by research staff.

### Sample Size

An a priori power calculation indicated that 48 participants were needed per group to detect an estimated difference of HbA1c of 0.65%, assuming a significance level of 5% (two-tailed), a standard deviation of 1.4, and a statistical power of 80%. We overenrolled to allow for attrition, setting our final recruitment target at 65 participants per group.

### Randomization

A random number sequence was generated using a random number-generating program without constraints [28]. After the sequence was generated by the research coordinator, a research assistant with no connection to the trial sealed the sequence in individual, opaque envelopes and numbered each based on sequence generation. Once a candidate participant consented and their HbA1c was verified as meeting the inclusion criteria, the next envelope was opened (in sequence) to ascertain group allocation, and the health coaching intervention commenced. Patient and coach blinding was impossible as participants readily identified receipt of a mobile phone with experimental group participation and the absence of receipt with control group participation.

### Statistical Analysis

Data were double entered by 2 independent research assistants to ensure accuracy. Baseline characteristics between intervention and control groups were compared for differences using independent samples *t* tests for continuous variables and chi-square for dichotomous variables. Primary outcome comparison was conducted with an independent samples *t* test using per-protocol and intention-to-treat analyses (last observation carried forward [LOCF]). Secondary outcome comparisons were conducted solely using per-protocol comparisons with a factorial repeated-measures analysis of variance (ANOVA). Data were analyzed using SPSS 21.0 (IBM Corp, Armonk, NY, USA).

## Results

### Overview

**Figure 6:**
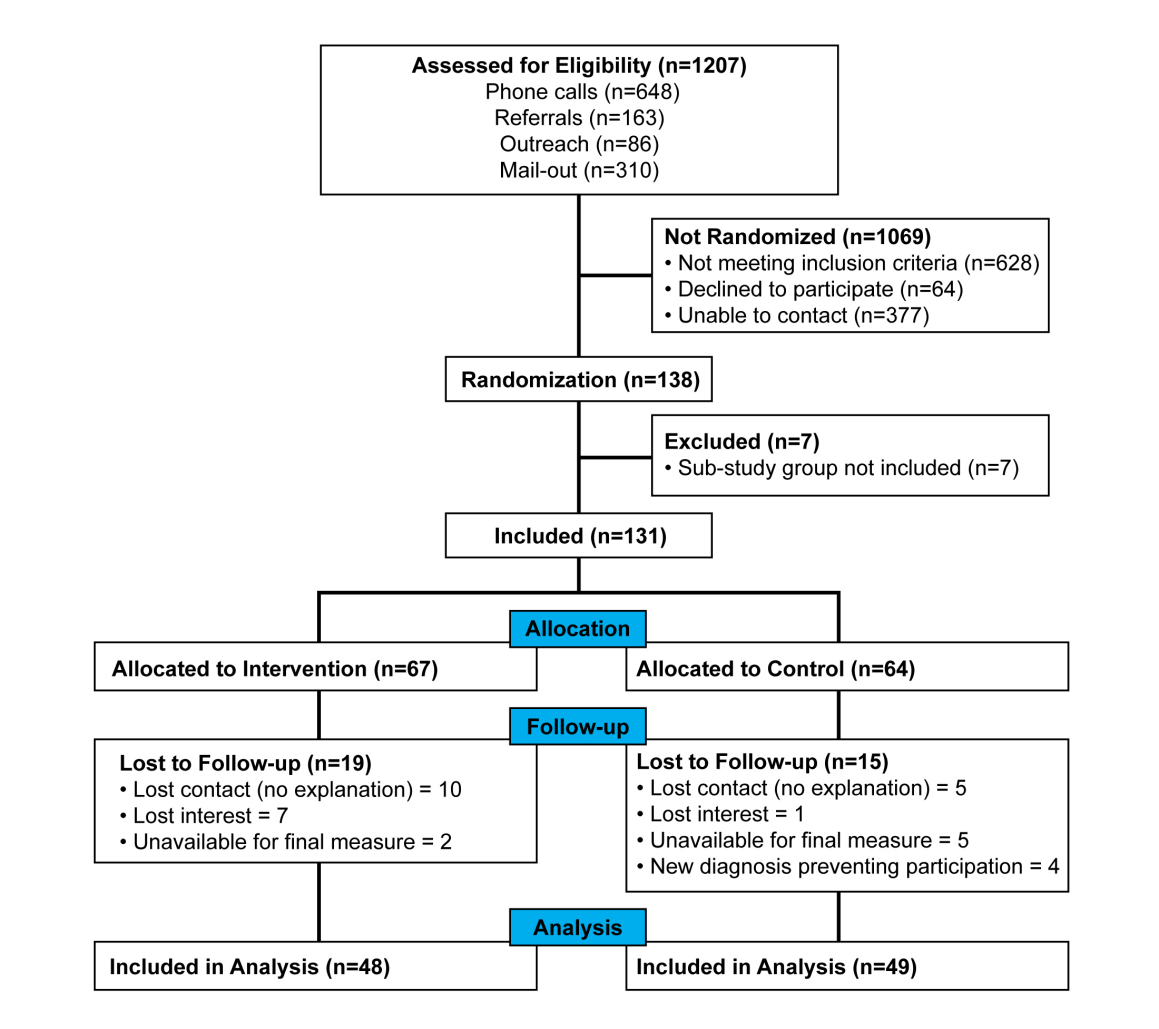
Flowchart of enrollment.

Between March 2012 and October 2013, 138 participants were recruited; 67 were randomized to the experimental arm and 64 to the control arm (7 were excluded for substudy analysis) as seen in the CONSORT diagram (Figure 6). A majority of participants (57/97, 59%) had not completed postsecondary education and 35 out of 97 (36%) were unemployed, while a total of 64 out of 97 (66%) reported household incomes of Can $50,000 or less. A majority of participants were recruited from Site Number 1 (90/97, 93%) and were female (70/97, 72%). Of the 131 participants included in the study, there were 34 dropouts (26%), with 19 out of 67 (28%) from the intervention group and 15 out of 64 (23%) from the control group. Independent samples *t* tests indicated no statistically significant differences between dropouts and trial completers for HbA1c or for demographic variables. Final per-protocol analysis included 97 participants out of 131 (74%), with 48 in the intervention group and 49 in the control group. Of the measures collected, differences at baseline between groups were only detected for the SF-12 Mental Health Composite Scores. Of the 48 participants allocated to the mobile phone group, mobile phone use data indicated that 39 out of 48 participants (81%) used the CWP with consistency to communicate with their health coach and track various health measures (eg, blood glucose, food, and/or exercise).

### Hemoglobin A1c

Independent samples *t* tests indicated no significant between-group differences in HbA1c from baseline to 6 months when analyzed with intention-to-treat (*P*=.48) and per-protocol (*P*=.83) principles (Table 2).

**Table 2:** Independent samples *t* test measuring differences in HbA1c levels from baseline to 6 months.

Type of analysis	n	Intervention group, mean (SD)	Control group, mean (SD)	Difference	*P* (two-tailed)
HbA1c: per protocol	97	-0.815 (1.050)	-0.759 (1.390)	0.055	.83
HbA1c: intention to treat	131	-0.642 (1.040)	-0.974 (1.400)	0.152	.48

Results from a repeated-measures ANOVA indicated trends for between-group HbA1c differences in a per-protocol analysis—*F*_1,89_=3.022, *P*=.09 (Table 3).

**Table 3:** Between-group analysis of variance measuring differences in HbA1c levels.

Type of analysis	n	Type II sum of squares	df	Mean square	*F*_1,89_	*P*	Partial eta squared
HbA1c: per protocol	97	3.004	1	3.004	3.002	.09	.034
HbA1c: intention to treat	131	1.463	1	1.463	1.142	.29	.009

These differences reflected significant HbA1c within-group reductions from baseline to 6 months in the intervention group—0.84% (9.2 mmol/mol), 95% CI 0.46-1.17; *P*=.001—and in the control group—0.81% (8.9 mmol/mol), 95% CI 0.41-1.11; *P*=.001—(Table 4), and a significantly greater reduction for the intervention group versus the control group at the 3-month follow-up (*P*=.03; Table 5).

**Table 4:** Change in HbA1c levels by group.

Measurement time point		Intervention group			Control group		
		n	Mean % (SD or 95% CI)	Total value (mmol/mol)	n	Mean % (SD or 95% CI)	Total value (mmol/mol)
**HbA1c included in** *t* **test (n=97)**							
	Baseline, mean (SD)	48	8.69 (1.32)	71.5	49	8.89 (1.30)	73.7
	6 months, mean (SD)	48	7.88 (1.17)	62.6	49	8.13 (1.27)	65.4
	Change from baseline to 6 months, mean (95% CI)	48	0.82 (0.46-1.17)^a^	8.9	49	0.76 (0.41-1.11)^a^	8.3
**HbA1c included in ANOVA** ^b^ **(n=89)**							
	Baseline, mean (SD)	45	8.60 (1.19)	70.5	44	8.88 (1.32)	73.6
	3 months, mean (SD)	45	7.74 (1.06)	61.1	44	8.26 (1.16)	66.8
	6 months, mean (SD)	45	7.76 (1.00)	61.3	44	8.07 (1.29)	64.7
	Change from baseline to 3 months, mean (95% CI)	45	0.86 (0.47-1.26)^a^	9.4	44	0.62 (0.23-1.03)^a^	6.8
	Change from baseline to 6 months, mean (95% CI)	45	0.84 (0.38-1.26)^a^	9.2	44	0.81 (0.34-1.28)^a^	8.9

^a^Significant at the *P*=.001 level.

^b^Analysis of variance (ANOVA).

A data discrepancy was detected during the repeated-measures ANOVA as 3 participants in the intervention group and 5 in the control group were not assessed at 3 months but were evaluated at 6 months. They had either refused the 3-month testing or their family physicians failed to provide their test results. Subsequent *t* tests indicated a lesser reduction in HbA1c (baseline to 6 months) for the controls lacking the 3-month data versus completers (*P*=.03). There were no differences in HbA1c (baseline to 6 months) for intervention participants lacking 3-month data versus those with complete data. Furthermore, no significant differences were found in baseline HbA1c levels for either intervention or controls participants with or without a 3-month HbA1c measure.

**Table 5:** HbA1c values for participants with and without 3-month measurements.

Measurement	Intervention group			Control group		
	3-month measure absent (n=3)	3-month measure present (n=45)	*P*	3-month measure absent (n=5)	3-month measure present (n=44)	*P*
Baseline HbA1c, mean % (SD)	9.97 (2.64)	8.60 (1.19)	.47	8.92 (1.19)	8.88 (1.33)	.95
Total HbA1c value (mmol/mol)	85.5	70.5		74	73.6	
Change in HbA1c (6 month-baseline), mean % (SD)	-0.40 (0.46)	-0.84 (1.08)	.47	-0.28 (0.19)	-0.81 (1.45)	.03

Table 6 shows that the HbA1c trend differences indicated with the repeated-measures ANOVA—*F*_1,89_=3.022, *P*=.09—were due to the greater reduction of HbA1c at 3 months in the intervention versus control group. This between-group difference disappeared at 6 months with gains in the control group, and no further gains in the intervention group.

**Table 6:** Time-point comparison of HbA1c levels for intervention versus control groups.

Time point	Between-group difference of % HbA1c (95% CI)	*P*
Baseline	0.280 (-0.250 to 0.810)	.30
3 months	0.515 (0.500 to 0.990)	.03
6 months	0.308 (-0.180 to 0.800)	.21

### Secondary Outcomes: Body Composition

We detected significant reductions in body weight (1.22 kg, 95% CI 0.35-2.08; *P*=.006) and waist circumference (2.23 cm, 95% CI 0.53-3.93; *P*=.01) in the intervention group, while the control group had no change. There were no significant changes in BMI in either group (Table 7).

### Secondary Outcomes: Psychometric Questionnaires

A significant number of trial completers chose not to complete psychometric questionnaires at follow-up, resulting in their baseline outcomes being omitted from additional analyses (Table 7). Comparison of the baseline psychometric outcomes of completers and noncompleters indicated no significant differences.

Within-group, pre/post improvements in life satisfaction were detected in the intervention (+3.72, 95% CI 1.50-5.94; *P*=.001) and control groups (+3.77, 95% CI 1.30-6.24; *P*=.003) (Satisfaction with Life Scale). Similar improvements for both intervention and control groups were detected in the Hospital Anxiety and Depression Scale (HADS) depression subscale (-1.81, 95% CI -2.81 to -0.81; *P*=.001; -1.70, 95% CI -2.73 to -0.67; *P*=.002), and the Physical Composite Score of the SF-12 (+2.69, 95% CI 0.21-5.17; *P*=.03; +2.92, 95% CI 0.24-5.60; *P*=.03) (Table 7), although the control group demonstrated a significantly reduced HADS anxiety subscale score (-1.50, 95% CI -2.73 to -0.27; *P*=.02), while the intervention group did not (-1.12, 95% CI -2.29 to 0.05; *P*=.06) (Table 7). Significant between-group differences were found at the 6-month follow-up for negative affect (negative affect subscale of the Positive and Negative Affect Schedule [PANAS]) (+5.27, 95% CI 1.51-9.04; *P*=.007) favoring the intervention group (Table 7).

**Table 7:** Baseline, follow-up, and change values for all secondary outcomes.

Variable by group		n	Baseline, mean (SD or 95% CI)	6-month follow-up, mean (SD or 95% CI)	Change, mean (95% CI)	*P*
**Weight (kg)**						
	Intervention	41	93.66 (20.23)	92.44 (20.24)	-1.22 (0.35-2.08)^a^	.006
	Control	39	98.76 (24.02)	99.21 (24.77)	+0.45 (-1.33 to 0.44)	.32
	Difference between groups		5.10 (-4.78 to 14.98)	6.76 (-3.29 to 16.81)		
	*P*		.31	.18		
**Waist circumference (cm)**						
	Intervention	40	112.11 (14.50)	109.88 (14.82)	-2.23 (0.53-3.93)^a^	.01
	Control	37	113.88 (17.04)	114.00 (18.12)	+0.122 (-1.89 to 1.64)	.89
	Difference between groups		1.78 (-5.39 to 8.94)	4.13 (-3.36 to 11.62)		
	*P*		.62	.28		
**Body mass index (kg/m** ^**2**^ **)**						
	Intervention	39	33.74 (6.70)	33.53 (6.80)	-0.21 (-0.24 to 0.66)	.35
	Control	36	37.00 (7.92)	37.21 (8.22)	-0.21 (-0.68 to 0.25)	.37
	Difference between groups		3.26 (-0.11 to 6.63)	3.69 (0.22-7.15)^a^		
	*P*		.06	.04		
**Satisfaction with Life**						
	Intervention	32	20.50 (7.71)	24.22 (6.33)	+3.72 (1.50-5.94)^b^	.001
	Control	26	18.04 (7.01)	21.81 (7.15)	+3.77 (1.30-6.24)^b^	.003
	Difference between groups		-2.46 (-1.46 to 6.38)	-2.41 (-1.14 to 5.96)		
	*P*		.21	.18		
**HADS** ^c^ **: anxiety subscale**						
	Intervention	33	7.39 (4.53)	6.27 (4.18)	-1.12 (-2.29 to 0.05)	.06
	Control	30	9.50 (4.49)	8.00 (5.06)	-1.50 (-2.73 to -0.27)^a^	.02
	Difference between groups		2.11 (-0.17 to 4.39)	1.73 (-0.60 to 4.06)		
	*P*		.07	.14		
**HADS: depression subscale**						
	Intervention	32	6.25 (3.99)	4.44 (3.32)	-1.81 (-2.81 to -0.82)^b^	.001
	Control	30	7.77 (4.06)	6.07 (4.38)	-1.70 (-2.73 to -0.67)^b^	.002
	Difference between groups		1.52 (-0.53 to 3.56)	1.63 (-0.34 to 3.60)		
	*P*		.14	.10		
**PANAS** ^d^ **: positive affect subscale**						
	Intervention	30	34.43 (8.46)	36.03 (7.65)	+1.60 (-1.00 to 4.20)	.22
	Control	27	31.22 (10.29)	31.67 (9.71)	+0.44 (-2.30 to 3.18)	.75
	Difference between groups		-3.21 (-1.77 to 8.19)	-4.37 (-0.25 to 8.98)		
	*P*		.20	.06		
**PANAS: negative affect subscale**						
	Intervention	31	16.58 (7.85)	14.55 (5.03)	-2.03 (-4.87 to 0.80)	.16
	Control	28	20.39 (9.57)	19.82 (9.04)	-0.57 (-3.55 to 2.41)	.70
	Difference between groups		3.81 (-0.73 to 8.36)	5.27 (1.51-9.04)^a^		
	*P*		.10	.007		
**SF-12** ^e^ **: Physical Composite Score**						
	Intervention	34	42.89 (8.69)	45.57 (7.54)	+2.69 (0.21-5.17)^a^	.03
	Control	29	41.63 (10.08)	44.55 (10.89)	+2.92 (0.24-5.60)^a^	.03
	Difference between groups		1.25 (-3.48 to 5.98)	1.02 (-3.65 to 5.68)		
	*P*		.60	.66		
**SF-12: Mental Composite Score**						
	Intervention	34	47.74 (11.11)	50.22 (10.29)	+2.48 (-1.10 to 6.05)	.17
	Control	29	41.68 (11.82)	44.50 (10.15)	+2.82 (-1.05 to 6.69)	.15
	Difference between groups		6.06 (0.28-11.85)^a^	5.72 (0.56-10.89)^a^		
	*P*		.04	.03		

^a^The change is significant, *P*<.05.

^b^The change is significant, *P*<.005.

^c^Hospital Anxiety and Depression Scale (HADS).

^d^Positive and Negative Affect Schedule (PANAS).

^e^Short Form Health Survey-12 (SF-12).

## Discussion

### Principal Findings

Personalized health coaching with and without the provisions of mobile phone and related software support was assessed in a predominantly lower-SES population with poorly controlled T2DM. A total of 45% of participants reported household incomes of Can $25,000 or less, qualifying them as living at or beneath the Canada poverty line [29], while an additional 20.9% of participants reported household incomes between Can $25,000 and Can $50,000. Our findings suggest clinically significant within-group reductions in HbA1c in both groups but no significant between-group differences in HbA1c from baseline to 6 months according to per-protocol (*P*=.83) and intention-to-treat (LOCF) (*P*=.48) analyses.

**Figure 7:**
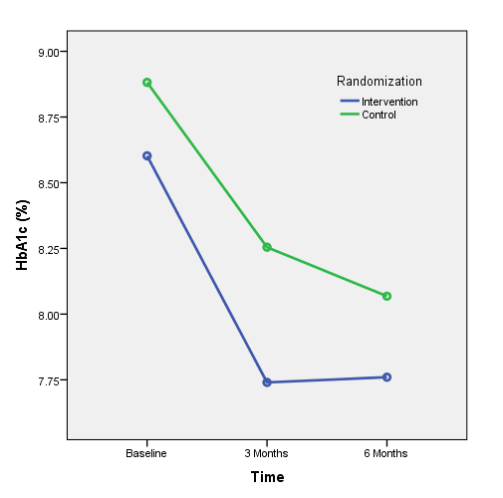
HbA1c levels for the control and intervention groups over time.

There was, however, a significant between-group difference in HbA1c at the 3-month time point (0.52%, *P*=.03) favoring the mobile phone-assisted group, although this difference was not statistically significant at 6 months because the control group’s mean HbA1c reduction improved between 3 and 6 months while the intervention group’s HbA1c level remained stable (Figure 7). This result indicates that clinically significant HbA1c reductions occurred at a faster rate with HC and mobile phone support than with solely HC support. The repeated-measures ANOVA analysis of three time points was affected by missing data; however, all missing control participants had no HbA1c reductions, resulting in an increased mean difference in remaining controls necessitating a larger effect size in the experimental condition to reflect a significant difference. Observed weight and waist circumference differences also suggested comparative benefits for the mobile phone-assisted group versus controls. These included significant reductions in weight and waist circumference in the mobile phone group which appeared to be related to the food photo-journaling function of the CWP. By reviewing photographs of their meals, participants could reflect on portion size and nutritional value in discussion *with* the health coach. These photo-stimulated "teachable moments" appeared to improve dietary choices more than was evident in the health coach-only group. Those in the mobile phone group also subjectively reported value in photographing meals and recording glucose levels in response to in-depth semistructured interviews [19]. Reductions in negative affect are likely linked to intervention participants feeling fundamentally connected in their health-focused program as their mobile phone became a constant symbol of being able to access a genuinely concerned person (24 hours a day/7 days a week) whose sole purpose was to help address health concerns. This feeling of health coach connectedness was a principal theme in the qualitative analyses of participant interviews [19].

Lower-SES populations confront higher mortality risks than equivalent higher-SES populations [6]. Due to a variety of challenges to health maintenance, individuals from lower-SES communities have poorer health status *and* use health care services more reactively [9]. They are also more likely to suffer from mental health conditions [30], *but* less likely to access mental health service resources [31]. Our results indicate that psychological well-being within the overall sample improved from baseline to 6-month follow-up, specifically demonstrated in outcomes on the Satisfaction with Life and the Hospital Anxiety and Depression Scales. As both groups communicated at least a once per week with their HCs, these interactions appeared sufficient for improvements in self-reported mood. Although differences in our primary outcome (HbA1c level) were only trending toward significant between-group differences, significant differences appeared in other markers of basic health (ie, weight and waist circumference), and in the negative affect subscale of the PANAS. Once again, those who used the mobile phone subjectively reported value in photographing meals and recording blood glucose levels when responding to in-depth semistructured interviews [19].

The Connected Wellness Platform enabled self-monitoring and health coach interactions with intervention participants, providing a cloud-based platform for mobile phone-based health management. This system provided secure, two-way communication between client and health coach, with mobile phone data entry on relevant behaviors entered manually. While the restriction to manual entry was not ideal, Bluetooth functionality for glucometers and pedometers was not yet integrated into the system during the trial. Other chronic disease management systems with similar features have been tested for usability and functionality. Notably, Martinez-Millana et al [32] comprehensively tested a diabetes management system with 30 patients and assessed the speed accuracy of tracking with several Bluetooth-enabled devices (ie, glucometers and pedometers) and their performance with a variety of mobile phones and network connections. Although we did not focus on the same performance analysis criteria during this trial, the CWP went through multiple upgrades during the pilot trial [3], ensuring smoother functionality and a more refined user interface (Figures 2-5) for the RCT. Detailed user experience with the CWP was collected using semistructured interviews and is reported in a full-length article [19]. CWP-user data logs were also extracted and analyzed with data mining methods to evaluate more finely tuned associations between app use and clinical outcomes (in a submitted manuscript).

Careful titrations of health coach interventions, typically measured by the frequency and duration of patient-coach interactions, are important elements in determining the optimal HC contact for eliciting improved health at minimal cost. With too little interaction, HC interventions risk insignificant or unsustainable health improvements, while too much interaction results in overly expensive implementation. As such, studies using multiple intervention intensities are necessary to ultimately determine appropriate contact level. Although we did not specify a minimum-maximum intervention intensity during the trial (providing weekly contact was maintained), the mean interaction intensity was 38 min/week (SD 25). In both intervention and control conditions, significant improvements in HbA1c levels and psychological functioning were found. The mobile technology appeared useful in engaging participants more quickly such that significantly greater HbA1c reductions were evident at 3 months (compared to controls), which may have cost-effectiveness implications as these gains were stabilized and evident at 6 months, although additional improvements in controls ultimately erased the 3-month differences. While the gains made at 3 months were sustained at 6 months (in the intervention group), there is no evidence that gains made in either group were sustained beyond the 6-month follow-up.

A unique feature of this study was the enhanced usual care that at least partly explains gains achieved by both control and intervention participants. The BCCHC site maintained a clinical exercise program that was several yards from the primary care physician and diabetes education team offices, symbolizing the importance of exercise in health maintenance, while serving patients in need. Moreover, the program provided T2DM patients with education, exercise prescription, and monitoring, which included the assessment of blood glucose levels before and after every supervised exercise session. This supported patients in recognizing the benefits of exercise in blood glucose regulation, and helped encourage adoption of home-based exercise programs. Since the HCs in this trial were all certified exercise specialists (through the Canadian Society for Exercise Physiology), exercise prescription was undertaken safely, with no adverse events, according to the highest evidence-based standards. A total of 23 intervention patients and 22 control patients participated in the Exercise Education Program. Although we might have included a control condition that did not access the EEP, the EEP was adopted as usual care at BCCHC and denial of access would have been unethical.

### Limitations

As with any behavioral intervention, motivations to participate introduce potential biases as those who met inclusion criteria but declined to participate represent an unstudied population. This limits the generalizability of the intervention [33]. As well, the comparison group received health coach support (without mobile monitoring) as opposed to usual care. Not only did this enable a more clear understanding of the effect of electronic monitoring of health behavior on clinical outcomes, pilot trial findings suggested a usual care control condition (ie, no health coaching) would result in an unacceptably high attrition rate in the controls. The lack of between-group differences at 6 months may be due to other, more complex factors. For example, since health coaches were randomly assigned to participants in both arms, it is possible that more effort was expended in coaching the mobile phone-assisted arm. However, since the effect size of HbA1c reduction was similar across groups, this was unlikely. Furthermore, there could have been bias in the opposite direction, with health coaches expending more effort in assisting the behavior change of control participants since these controls did not have the support of the mobile phone interactions. Also, although it would have been ideal to compare multiple glucose measures (eg, random blood sugar, fasting blood sugar), it was not possible at the participating sites. We were limited to reliable access only to HbA1c blood tests. We recognize, with other researchers, that glucose regulation is more complex than what is solely indicated in HbA1c assessment.

### Conclusions

Although this trial did not indicate a significant between-group difference in improved glucoregulation, there were overall clinical and statistically significant improvements in HbA1c for participants in both health-coached groups. Given the pragmatic trial design, our findings suggest health coaching in primary care can improve the glucose management of poorly controlled T2DM in lower-SES community residents. It is evident that using mobile phones to further connect patients to health coaches and monitor health behaviors can lead to faster reductions in HbA1c, which may have specific benefits for cost savings and quality of life. Further research comparing health-coaching interventions of different contact intensities, using wearable biomonitoring devices, and using a true waitlist/control group will help evaluate health coach intervention effectiveness, as well as long-term adherence levels and cost/benefit results.

## Abbreviations

ANOVA: analysis of variance
BCCHC: Black Creek Community Health Centre
BMI: body mass index
CSEP: Canadian Society for Exercise Physiology
CWP: Connected Wellness Platform
EEP: Exercise Education Program
HADS: Hospital Anxiety and Depression Scale
HbA1c: glycated hemoglobin/hemoglobin A1c
HC: health coach
HRQOL: health-related quality of life
LOCF: last observation carried forward
NYFHT: North York Family Health Team
OHIP: Ontario Health Insurance Program
PANAS: Positive and Negative Affect Schedule
RCT: randomized controlled trial
SES: socioeconomic status
SF-12: Short Form Health Survey-12
T2DM: type 2 diabetes mellitus

## Appendix

Multimedia Appendix 1 CONSORT-eHEALTH (V 1.6.1) - submission/publication Form.
